# Unsupervised Neural Beamforming for Uplink MU-SIMO in 3GPP-Compliant Wireless Channels †

**DOI:** 10.3390/s26020366

**Published:** 2026-01-06

**Authors:** Cemil Vahapoglu, Timothy J. O’Shea, Wan Liu, Tamoghna Roy, Sennur Ulukus

**Affiliations:** 1 Department of Electrical and Computer Engineering, University of Maryland, College Park, MD 20742, USA; cemilnv@umd.edu; 2 DeepSig Inc., Arlington, VA 22203, USA; tim@deepsig.io (T.J.O.); wliu@deepsig.io (W.L.); tamoghna.roy@deepsig.io (T.R.)

**Keywords:** unsupervised learning, uplink beamforming, MIMO, neural beamforming, throughput maximization

## Abstract

Beamforming is highly significant for the physical layer of wireless communication systems, for multi-antenna systems such as multiple input multiple output (MIMO) and massive MIMO, since it improves spectral efficiency and reduces interference. Traditional linear beamforming methods such as zero-forcing beamforming (ZFBF) and minimum mean square error (MMSE) beamforming provide closed-form solutions. Yet, their performance drops when they face non-ideal conditions such as imperfect channel state information (CSI), dynamic propagation environment, or high-dimensional system configurations, primarily due to static assumptions and computational limitations. These limitations have led to the rise of deep learning-based beamforming, where data-driven models derive beamforming solutions directly from CSI. By leveraging the representational capabilities of cutting-edge deep learning architectures, along with the increasing availability of data and computational resources, deep learning presents an adaptive and potentially scalable alternative to traditional methodologies. In this work, we unify and systematically compare our two unsupervised learning architectures for uplink receive beamforming: a simple neural network beamforming (NNBF) model, composed of convolutional and fully connected layers, and a transformer-based NNBF model that integrates grouped convolutions for feature extraction and transformer blocks to capture long-range channel dependencies. They are evaluated in a common multi-user single input multiple output (MU-SIMO) system model to maximize sum-rate across single-antenna user equipments (UEs) under 3GPP-compliant channel models, namely TDL-A and UMa. Furthermore, we present a FLOPs-based asymptotic computational complexity analysis for the NNBF architectures alongside baseline methods, namely ZFBF and MMSE beamforming, explicitly characterizing inference-time scaling behavior. Experiments for the simple NNBF are performed under simplified assumptions such as stationary UEs and perfect CSI across varying antenna configurations in the TDL-A channel. On the other hand, transformer-based NNBF is evaluated in more realistic conditions, including urban macro environments with imperfect CSI, diverse UE mobilities, coding rates, and modulation schemes. Results show that the transformer-based NNBF achieves superior performance under realistic conditions at the cost of increased computational complexity, while the simple NNBF presents comparable or better performance than baseline methods with significantly lower complexity under simplified assumptions.

## 1. Introduction

Beamforming is a fundamental signal processing technique in the physical layer of wireless communication systems, which is designed to spatially direct transmitted or received signals in order to improve signal quality, spectral efficiency, and reduce interference. It has significance in multi-antenna systems such as multiple input multiple output (MIMO) and massive MIMO systems, where beamforming enables spatial multiplexing and user separation.

Conventional linear beamforming methods include zero-forcing beamforming (ZFBF) and minimum mean square error (MMSE) beamforming, which are designed based on perfect or estimated channel state information (CSI) and aim to optimize signal-to-noise ratio (SNR) and interference suppression [1,2]. These methods have been extensively analyzed for their simplicity and closed-form solutions [3,4,5,6]. However, their efficacy degrades under imperfect CSI or when applied to high-dimensional systems [7,8]. There are numerous reasons for this: Most approaches assume static network conditions, leading to suboptimal performance in dynamic environments due to dependence on algorithm initialization [9,10]. In addition, they are constrained by computing complexities, which create a gap between theoretical analysis and real-time implementation [11].

To tackle these challenges, deep learning-based beamforming has emerged as a promising approach, enabling data-driven models to learn complex beamforming strategies directly from CSI by employing dynamic spectrum data rather than depending on predefined policies [12,13]. By considering representational capabilities of cutting-edge deep learning architectures along with advancements in computational and data resources, the importance of deep learning-based methodologies becomes clearer [14].

Research efforts have shown the ability to generalize under realistic impairments such as channel estimation error, user mobility, and limited feedback. They intend to approximate or outperform conventional beamforming techniques, particularly in scenarios where traditional approaches encounter drawbacks. Ref. [15] proposes a supervised learning framework that consists of convolutional neural networks (CNNs) to design hybrid beamforming and channel estimation while reducing the complexity and providing the robustness on the receiver without instantaneous CSI feedback. The authors of [16] suggest that signal processing problems in wireless communications such as beamforming design and power control can be evaluated from a learning-based perspective. They consider the input and output of an optimization algorithm, specifically the weighted MMSE algorithm, as an unknown nonlinear mapping to be approximated by a deep neural network (DNN). In [17], a deep fully convolutional neural network is introduced for downlink beamforming design, leveraging uplink channel estimates in a supervised manner. Additionally, ref. [18] proposes three beamforming neural networks based on CNNs and expert knowledge to utilize the known structure of optimal solutions for the signal-to-interference-plus-noise ratio (SINR) balancing problem, power minimization problem, and sum-rate maximization problem. According to the expert knowledge regarding the known structure of downlink beamforming vectors as a closed form solution indicated by [19], the authors leverage the principle that the power minimization problem, subject to SINR constraints, yields beamforming vectors that meet the SINR targets specified as lower bound constraints, while utilizing the minimal amount of power. Therefore, the resultant beamforming vectors lie within the feasible set of the sum-rate maximization problem under a power constraint, also turning them into an optimal solution for the sum-rate maximization problem, as they utilize the minimal power. Yet, the calculation of downlink beamforming vectors for sum-rate maximization via a known structure necessitates matrix inversion, which may impose a computational burden on a real-time processing system, particularly when the system has a substantial number of antennas as in massive MIMO. Ref. [20] proposes a joint supervised learning framework for channel prediction, power optimization, and transmit beamforming prediction in the downlink channel. It also exploits the known parameterized structure of a beamforming solution for sum-rate maximization as in [18].

All aforementioned works utilize a supervised learning approach, which requires labeled data that is often impractical to obtain in real scenarios, while some also incur additional computational burden for massive MIMO systems. The authors of [21] propose a fast beamforming design method using unsupervised learning for sum-rate maximization under total power constraint. The proposed model is computationally efficient for end-to-end beamforming design. However, the channel matrix is represented using independent and identically distributed complex Gaussian entries for dataset generation by modeling a Rayleigh flat fading channel model. Consequently, it is unable to represent realistic channel characteristics, including frequency selectivity, delay spread, and Doppler spread, among others. In addition to function approximation via DNNs, some studies approximate iterative optimization algorithms through deep unfolding methods [22]. While applicable to various wireless communication tasks [23,24], these approaches may also impose considerable computational costs, since matrix inversion or singular value decomposition (SVD) can still be required.

The contributions of this work can be summarized as follows. First, we unify two unsupervised Neural Network Beamforming (NNBF) architectures, simple NNBF [25] and transformer-based NNBF [26], under a common multi-user single input multiple output (MU-SIMO) system model and sum-rate maximization objective, facilitating a systematic and direct comparison that is not provided in prior studies. Second, we provide the asymptotic FLOPs-based computational complexity analysis for simple NNBF and transformer-based NNBF, alongside conventional beamforming techniques ZFBF and MMSE beamforming. This analysis specifically characterizes the inference-time computational complexity of the considered NNBF architectures by identifying the dominant asymptotic scaling behavior with respect to the number of receive antennas, number of users, and OFDM resources. Both NNBF models are trained using an unsupervised learning approach and are evaluated through comprehensive simulations under 3GPP-compliant channel models [27], with ZFBF and MMSE beamforming serving as baseline techniques.

For simple NNBF, it is assumed that perfect CSI is available at the receiver when user equipments (UEs) are stationary with near-zero maximum Doppler shift under the TDL-A channel model. The results show that simple NNBF performs comparably to or better than MMSE baseline, while it consistently outperforms ZFBF across all SNR regimes. Furthermore, both theoretical and empirical evidence demonstrate that simple NNBF offers a computationally efficient framework compared to the baseline techniques.

Grouped convolutions offer parameter efficiency for extracting spatial and temporal features with minimal performance loss, making them valuable for resource-constrained environments [28,29]. Transformers have shown strong potential in capturing long-range channel dependencies and adapting beamforming strategies under dynamic network conditions through attention mechanisms [30,31,32,33,34]. Considering these advantages, the transformer-based NNBF architecture is designed by integrating both transformers and grouped convolutions. For transformer-based NNBF architecture, a more realistic scenario is considered in dense urban environments, which are modeled by the urban macro (UMa) channel. The UMa channel provides a realistic representation of urban wireless environments by capturing the key characteristics of dense urban deployments [27]. Moreover, it is considered that only imperfect CSI, acquired through channel estimation in the radio unit (RU), is accessible, when UEs can have substantial mobility with high maximum Doppler shift. In addition to spectral efficiency, block error rate (BLER) is considered as a performance metric for transformer-based NNBF. Experimental results across diverse configurations for varying UE mobilities, coding rates, and modulation orders demonstrate the superiority of the transformer-based NNBF over baseline methods ZFBF and MMSE beamforming. Moreover, the complexity analysis highlights a trade-off between the enhanced modeling capacity of transformer-based NNBF, enabled by attention mechanisms that capture long-range dependencies, and its computational cost under realistic channel conditions.

## 2. System Model and Problem Formulation

### 2.1. Uplink Multi-User SIMO (MU-SIMO) Setup

**Figure 1:**
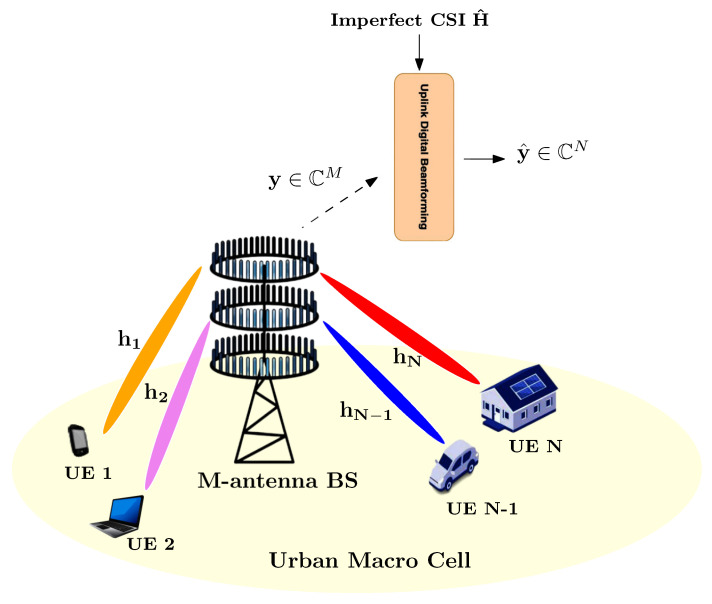
Uplink multi-user SIMO system in a dense urban environment, where single-antenna UEs transmit data streams on the same time/frequency resources and M-antenna BS apply digital beamforming on the received signal y.

We examine an uplink transmission scenario wherein *N* single-antenna UEs transmit data streams to a base station (BS) equipped with *M* receive antennas, as illustrated in Figure 1.

The uplink channel matrix is represented as $H=\left[h_{1}\;h_{2}\;\ldots \;h_{N}\right]\in C^{M\times N}$, where $h_{k}$ corresponds to the channel vector between UE *k* and BS. The received signal y can be expressed as

(1) $$\begin{array}{c} y=\sum_{i=1}^{N}h_{i}x_{i}+n \end{array}$$

where $x={\left[x_{1}^{H}\;x_{2}^{H}\;\ldots \;x_{N}^{H}\right]}^{H}\in C^{N}$ represents the transmitted signal, with each entry corresponding to the modulated data symbols sent by the UEs, satisfying $E[x_{k}^{H}x_{k}]=1,\;\forall k=1,\ldots ,N.$ Additionally, $n={\left[n_{1}^{H}\;n_{2}^{H}\;\ldots \;n_{M}^{H}\right]}^{H}\in C^{M}$ denotes the additive white Gaussian noise with i.i.d. entries $n_{l}∼\mathrm{CN}\left(0,\sigma^{2}\right),\;\forall l=1,\ldots ,M$.

Our system model includes a simple case with perfect CSI accessible at the BS and stationary UEs experiencing minimal maximum Doppler shift, as well as a scenario with imperfect CSI involving mobile UEs with significant maximum Doppler shift.

While we analyze both scenarios independently, the received signal in (1) is processed using beamforming weights $W=\left[w_{1}\;w_{2}\;\ldots \;w_{N}\right]\in C^{M\times N}$ to extract data symbols, ensuring that the power consumption of the beamforming weights adheres to the constraint $w_{k}^{H}w_{k}\leq 1,\forall k=1,\ldots ,N$. Specifically, $w_{k}\in C^{M}$ functions as the linear beamforming filter for determining the transmitted data symbol of UE *k*, with the objective of maximizing throughput while minimizing interference from other users.

(2) $$\begin{array}{cc} w_{k}^{T}y & =\sum_{i=1}^{N}w_{k}^{T}h_{i}x_{i}+w_{k}^{T}n \end{array}$$

### 2.2. Uplink Performance Improvement in O-RAN

The Open Radio Access Network (O-RAN) provides various fronthaul (FH) interface options, with the 7.2x functional split emerging as the industry-preferred configuration. This split separates the physical (PHY) layer into two parts: low-PHY functions including FFT, cyclic prefix removal, and in some configurations, channel estimation and beamforming, which are conducted in the Open Radio Unit (O-RU), and high-PHY functions such as decoding and, depending on deployment, channel estimation, beamforming, or equalization, which are executed in the Open Distributed Unit (O-DU) [35]. In Category A (Cat-A) configurations, the O-RU is limited to basic low-PHY operations, forwarding frequency-domain per-antenna IQ samples to the O-DU, which executes all further PHY operations including channel estimation and beamforming. On the other hand, Category B (Cat-B) RUs are capable of conducting uplink channel estimation and digital beamforming locally, hence minimizing fronthaul bandwidth and latency. Equalization generally takes place in the O-DU, although it may be optionally performed in the O-RU in tailored implementations.

The Uplink Performance Improvement (ULPI) specification was introduced by O-RAN Working Group 4 in order to improve uplink processing efficiency and mitigate fronthaul bandwidth limitations. ULPI is not an entirely new functional split; rather, it is an enhancement of the current 7.2x split, offering architectural improvements for a better functional distribution between the O-RU and O-DU in the uplink. Two types of ULPI were established: ULPI-A, enhancing Cat-A 7.2x, and ULPI-B, enhancing Cat-B 7.2x [36].

In ULPI-A, which builds upon Cat-A, the O-RU continues to transmit per-antenna frequency-domain IQ samples to the O-DU, incorporating improved compression and more relaxed latency requirements. Channel estimation, beamforming, and equalization are still executed centrally in the O-DU. ULPI-A reduces fronthaul overhead by implementing efficient quantization and compression techniques for FFT output data and pilot-related metadata.

In ULPI-B, which extends Cat-B, the O-RU also conducts uplink channel estimation and digital beamforming, which may include conventional techniques such as maximum ratio combining (MRC), zero-forcing (ZF), and MMSE beamforming, as well as more advanced learning-based beamforming techniques, subsequently transmitting user-separated beamformed streams to the O-DU. Equalization and partial demodulation may be optionally executed in the O-RU, facilitating the transmission of soft symbols or demodulated bits. This architecture enables minimal fronthaul bandwidth and latency, particularly in environments with high user density and massive MIMO arrays. Furthermore, RU-side autonomy enhances scalability by separating certain PHY-layer operations from the O-DU.

Overall, this distribution of uplink intelligence into the O-RU enhances system scalability, decreases latency, and facilitates increased uplink throughput, especially in dense and massive MIMO configurations. ULPI enhancements constitute a crucial advancement in achieving more distributed and efficient open RAN architectures. Uplink functional distributions for option 7.2x split and ULPI variants are summarized in Table 1.

**Figure 2:**
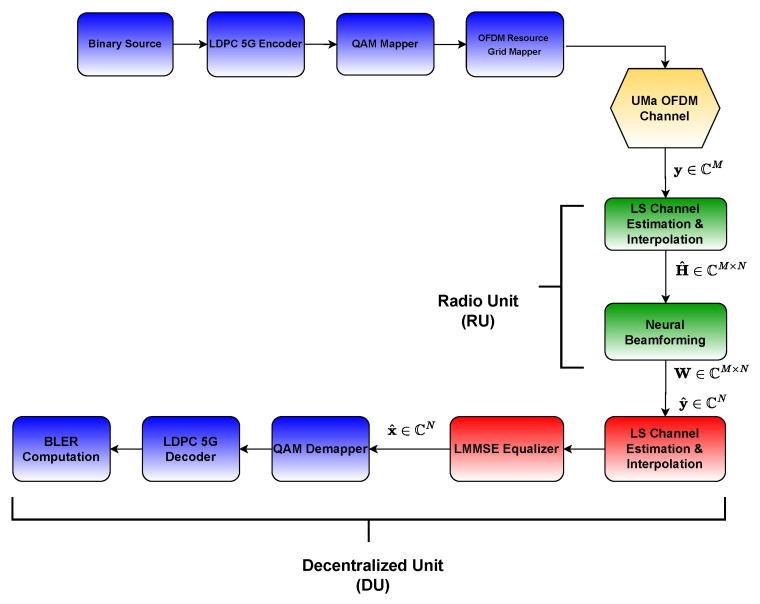
The block diagram of the system model.

In our work, it is assumed that ULPI-B is adopted. In our configuration, uplink channel estimation and beamforming are executed within the O-RU, while the O-DU is responsible for both uplink channel estimation and uplink equalization. Therefore, the uplink channel estimate $\hat{H}=\left[{\hat{h}}_{1}\;{\hat{h}}_{2}\;\ldots \;{\hat{h}}_{N}\right]\in C^{M\times N}$ is calculated to facilitate beamforming design directly within the RU, ensuring that all the necessary uplink processing tasks for beamforming, including the beamforming design itself, are efficiently managed locally within the RU. The block diagram of the system model, illustrating the RU-DU split, can be seen in Figure 2.

### 2.3. Beamforming Design for Sum-Rate Maximization

Our aim is to generate beamforming weights that optimize the total sum-rate for all UEs. The signal obtained for UE *k* subsequent to beamforming with $w_{k}$ can be restructured as

(3) $$\begin{array}{cc} {\hat{y}}_{k} & =w_{k}^{T}y\\ \; & =\underset{desired\;signal}{\underset{︸}{w_{k}^{T}h_{k}x_{k}}}+\underset{interfering\;signal}{\underset{︸}{\sum_{i=1,i\neq k}^{N}w_{k}^{T}h_{i}x_{i}}}+\underset{noise}{\underset{︸}{w_{k}^{T}n}} \end{array}$$

The initial term in (3) indicates the intended symbol for UE *k*, whereas the subsequent two terms indicate the inter-symbol interference (ISI) from other UEs and the receiver noise, respectively. SINR for UE *k*, $\gamma_{k}$, is expressed as

(4) $$\begin{array}{c} \gamma_{k}=\frac{|w_{k}^{T}h_{k}{|}^{2}}{\sum_{i=1,i\neq k}^{N}|w_{k}^{T}h_{i}{|}^{2}+E{\left|w_{k}^{T}n\right|}^{2}} \end{array}$$

Consequently, the beamforming design that is intended to maximize the sum-rate can be formulated as

(5) $$\begin{array}{cc} W^{*}=\underset{W}{arg max} & \;\sum_{i=1}^{N}\alpha_{i}\log\left(1+\gamma_{i}\right)\\ s.t. & \;\mathrm{tr}(W^{H}W)\leq N \end{array}$$

where $\alpha_{i}$ denotes the rate weighting factor for each UE *i*, which is also a trainable parameter within our framework, ensuring that $\sum_{i=1}^{N}\alpha_{i}=1$. The constraint $\mathrm{tr}(W^{H}W)\leq N$ imposes a total power budget proportional to the number of UEs. Alternatively, if a power constraint for each receive antenna is implemented, the power budget can be adjusted by a factor of *M*, where *M* represents the number of receive antennas. This differentiation enables adaptability in modeling various hardware constraints or fairness standards at the BS.

## 3. Proposed Deep Neural Networks

This section presents two deep neural network-based beamforming (NNBF) architectures designed to tackle the sum-rate maximization problem outlined in (5). Both networks have been designed to correlate the input, the frequency response of channel IQ data, represented by $\hat{H}$, to the output beamforming weights W as specified in the system model. In the remainder of the paper, *B* indicates the batch size, while *L* and *K* denote the number of OFDM symbols and subcarriers, respectively. To address diverse CSI quality, user mobility levels, and channel environments, we propose two architectural solutions designed for distinct deployment conditions.

- The simple NNBF architecture is intended for scenarios with perfect or near-perfect CSI. It assumes a tapped delay line (TDL) channel model and is well-suited for settings with stationary UEs and negligible Doppler shift. This architecture utilizes accurate channel information to learn beamforming weights in a computationally efficient way using a lightweight structure.
- The transformer-based NNBF architecture targets more realistic and challenging scenarios involving imperfect CSI and is assessed using the UMa channel model. It is especially appropriate for challenging deployment environments, including those with high user mobility and significant Doppler spread. By incorporating attention mechanisms, the architecture captures long-range dependencies across OFDM symbols and subcarriers, enabling robust beamforming performance in complex and dynamic environments.

### 3.1. Simple NNBF Architecture

**Figure 3:**
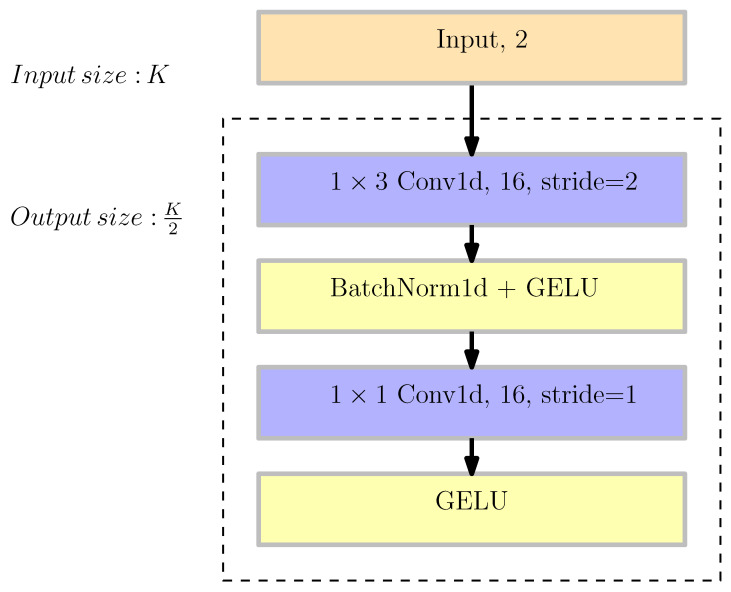
Basic block structure (dashed part) with 2 input channels and 16 output channels, BB(2,16).

The backbone of the simple NNBF architecture is composed of convolutional layers followed by batch normalization and activation layers, which are denoted by a basic block (BB) structure together, as shown in Figure 3.

The convolutional layers function on the frequency domain information obtained via the Fourier transform of channel tap data. Flat fading is presumed across time slots due to the maximum Doppler shift being set at 10 Hz. Consequently, variations in channel coefficients are restricted to disparities across subcarriers. We employ a CNN in our BB structure, in which 1D convolutions operate on the frequency dimension. To make the input data shape compatible, we reshape it as $\left(BNM,2,K\right)$, where *B* stands for the batch size of MU-SIMO channel matrices, and the depth dimension represents the IQ samples, while *K* represents the number of frequency components. Batch normalization facilitates faster convergence and reduces sensitivity to the initialization of network parameters. GELU serves as an activation function, demonstrating improved performance relative to RELU and ELU activations in various tasks, including computer vision, natural language processing, and speech tasks [37]. Furthermore, we increase the number of channels while reducing the dimensions of the feature map by half within the basic block structure. Taking into account the local correlations of physical channels in the frequency domain, an increase in network depth enhances latent space representation while facilitating a more concentrated analysis of local characteristics. This approach is widely utilized in computer vision tasks through prominent model architectures that enhance nonlinearity, allowing for the capture of intricate data relationships [38,39].

**Figure 4:**
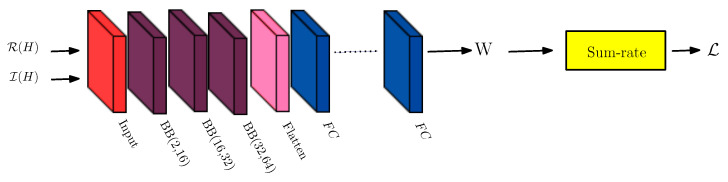
Simple NNBF architecture designed for scenarios with stable channel conditions, such as stationary UEs and minimal Doppler shift, leveraging perfect CSI and a lightweight structure to efficiently learn beamforming weights.

The simple NNBF architecture with the concatenation of basic block structures is illustrated in Figure 4. These blocks are characterized by prespecified input and output channel quantities. The flatten layer changes the output shape by concatenating depth dimension for all antenna pairs (n,m), where n=1,…,N and m=1,…,M. Then, the input shape of the first FC layer is (B,8NMK) as shown in Figure 4. The network output after fully connected (FC) layers is reshaped to have beamforming weights W. Furthermore, power normalization is performed for each receive antenna to satisfy the per-receive antenna power constraint.

### 3.2. Transformer-Based NNBF Architecture

**Figure 5:**
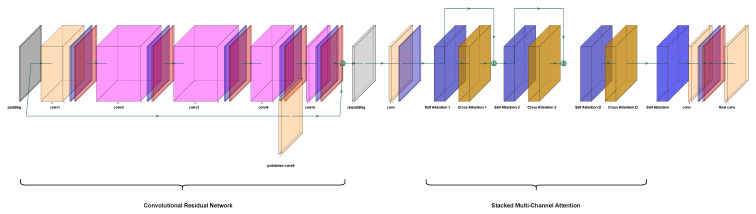
Transformer-based NNBF architecture composed of a Convolutional Residual Network and Stacked Multi-Channel Attention. Designed for dynamic channel conditions with high mobility and Doppler spread.

The proposed DNN architecture consists of two primary components: Convolutional Residual Network and Stacked Multi-Channel Attention as shown in Figure 5.

#### 3.2.1. ConvolutionalResidual Network

The first part of the model employs a convolutional residual network, which combines regular and grouped convolutions [40]. At this stage, the network receives the frequency response of imperfect channel estimate $\hat{H}$ as input, reshaped to dimensions (B,2MN,L,K); padding is first applied along (L,K) with mirror reflections of edge values.

This is followed by a regular convolution (orange “conv1” in Figure 5) and a sequence of grouped convolutions (vivid magenta layers in Figure 5). It enables the capture of local features effectively while enhancing efficiency through the grouped convolutions [40]. The number of groups for grouped convolutions is determined as the minimum of the number of input channels and the number of output channels. Moreover, residual connection is integrated to facilitate the gradient flow while pointwise convolution follows residual connection to consolidate the features from the residual path. All convolutional layers except pointwise convolution are followed by batch normalization and GELU activation function.

#### 3.2.2. Stacked Multi-Channel Attention

The second part of the model utilizes self attention and cross attention mechanisms to capture intra-channel relationships and inter-channel dependencies. In [41], it was shown that combining self attention and cross attention mechanisms can effectively capture contextual relationships within and between channels for speech recognition. Our proposed architecture demonstrates that self attention and cross attention mechanisms are also valuable for interference mitigation in 5G MIMO networks, specifically, in dense urban environments.

**Figure 6:**
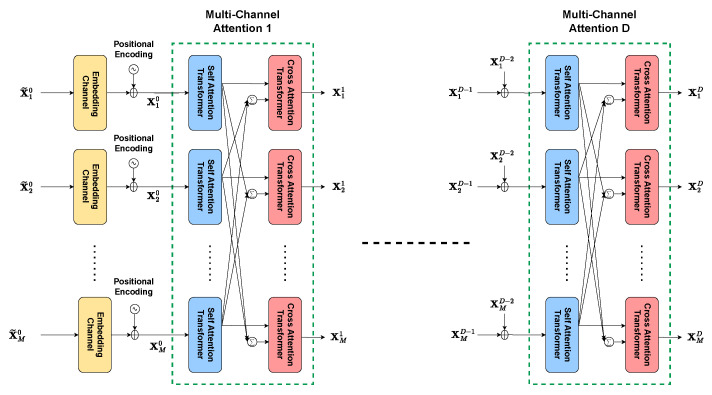
Stacked Multi-Channel Attention module to capture long-range dependencies across OFDM symbols and subcarriers, enabling robust beamforming performance under imperfect CSI.

Stacked multi-channel attention can be seen in Figure 6. It is the repeated sequence of self attention and cross attention transformer layers. Before the transformer layers, input features are divided into *M* chunks along the channel dimension, i.e., input of shape (B,depth,L,K) is split into *M* non-overlapping components, each with dimensions $\left(B,\frac{\mathrm{depth}}{M},L,K\right)$. Divided features are projected to dense embedding spaces. Channel embeddings are 1×1 unbiased convolutional layers followed by batch normalization. Positional encoding is employed on embedded space representations.

In the self attention transformer layer, there are two sublayers, as suggested by [42]. The first sublayer consists of scaled dot-product attention to compute the attention weights across time and frequency resources (L,K) to score over channel dimensions. Queries, keys, and values are computed by 1×1 convolution followed by GELU activation function. Specifically, for the *i*th embedding representation and *d*th self attention, they can be expressed as

(6) $$\begin{array}{cc} Q_{i,\mathrm{sa}}^{d} & =\mathrm{GELU}\left(X_{i}^{d-1}⊛F_{i,q}^{d}+b_{i,q}^{d}\right)\\ K_{i,\mathrm{sa}}^{d} & =\mathrm{GELU}\left(X_{i}^{d-1}⊛F_{i,k}^{d}+b_{i,k}^{d}\right)\\ V_{i,\mathrm{sa}}^{d} & =\mathrm{GELU}\left(X_{i}^{d-1}⊛F_{i,v}^{d}+b_{i,v}^{d}\right) \end{array}$$

where $F_{i,q}^{d}$, $F_{i,k}^{d}$, $F_{i,v}^{d}$$\in R^{\dim\times \dim}$ represent trainable convolutional filters while $b_{i,q}^{d}$, $b_{i,k}^{d}$, $b_{i,v}^{d}$$\in R^{\dim}$ are trainable bias parameters of *d*th self attention for input $X_{i}^{d-1}$. dim corresponds to number of input and output channels $\frac{\mathrm{depth}}{M}$. Then, corresponding self attention output is computed as

(7) $$\begin{array}{cc} A_{i,\mathrm{sa}}^{d} & =\mathrm{Softmax}\left(\frac{Q_{i,\mathrm{sa}}^{d}{\left(K_{i,\mathrm{sa}}^{d}\right)}^{T}}{\sqrt{\dim}}\right)V_{i,\mathrm{sa}}^{d} \end{array}$$

In the second sublayer of the self attention transformer, fully connected layers with a GELU activation function serve as the feed forward network, producing the final output of self attention transformer $Z_{i}^{d}$. Channel dimension is fed into the fully connected layers as features, i.e., input of second sublayer is reshaped as $\left(BLK,\frac{\mathrm{depth}}{M}\right)$. Additionally, residual connections and normalization are used for each sublayer as described in [42].

In the cross attention transformer layer, there are two sublayers similar to those in self attention. The first sublayer consists of scaled dot-product attention weights across time and frequency resources (L,K). Unlike self attention, keys and values are derived by performing 1×1 convolution on a weighted summation of other self attention outputs ${\bar{Z}}_{i}^{d}=\sum_{j=1,j\neq i}^{M}\beta_{j}^{d}Z_{j}^{d}$, where β parameters are trainable as well. The queries are computed through 1×1 convolution on the self attention output of the same input $Z_{i}^{d}$

(8) $$\begin{array}{cc} Q_{i,\mathrm{ca}}^{d} & =\mathrm{GELU}\left(Z_{i}^{d}⊛{\tilde{F}}_{i,q}^{d}+{\tilde{b}}_{i,q}^{d}\right)\\ K_{i,\mathrm{ca}}^{d} & =\mathrm{GELU}\left({\bar{Z}}_{i}^{d}⊛{\tilde{F}}_{i,k}^{d}+{\tilde{b}}_{i,k}^{d}\right)\\ V_{i,\mathrm{ca}}^{d} & =\mathrm{GELU}\left({\bar{Z}}_{i}^{d}⊛{\tilde{F}}_{i,v}^{d}+{\tilde{b}}_{i,v}^{d}\right) \end{array}$$

where ${\tilde{F}}_{i,q}^{d}$, ${\tilde{F}}_{i,k}^{d}$, ${\tilde{F}}_{i,v}^{d}$$\in R^{\dim\times \dim}$ represent trainable convolutional filters and ${\tilde{b}}_{i,q}^{d}$, ${\tilde{b}}_{i,k}^{d}$, ${\tilde{b}}_{i,v}^{d}$$\in R^{\dim}$ are trainable bias parameters of *d*th cross attention. Then, corresponding cross attention output is computed as

(9) $$\begin{array}{cc} A_{i,\mathrm{ca}}^{d} & =\mathrm{Softmax}\left(\frac{Q_{i,\mathrm{ca}}^{d}{\left(K_{i,\mathrm{ca}}^{d}\right)}^{T}}{\sqrt{\dim}}\right)V_{i,\mathrm{ca}}^{d} \end{array}$$

In the second sublayer of the cross attention transformer, fully connected layers with a GELU activation serve as the feed forward network, generating the final output of the *d*th multi-channel attention for the *i*th embedding input, denoted by $X_{i}^{d}$. Residual connections and normalization are applied to each sublayer, similarly to the approach in the self attention transformer. Additionally, multi-channel attention modules also have residual connections between each module.

Following the stacked multi-channel attention module, an additional self attention transformer is employed to compute the attention weights across antenna pairs (M,N). It shares the same structures as the one used in the multi-channel attention module. Finally, the network architecture concludes with regular convolutional layers to generate beamforming weights, denoted by $W_{nn}$.

### 3.3. Training Procedure

For both simple NNBF and transformer-based NNBF, unsupervised training is offered. The objective is to maximize the sum-rate across all UEs. Therefore, the loss function is defined according to the sum-rate maximization problem given in (5)

(10) $$\begin{array}{c} L\left(\theta ;\hat{H},W_{nn}\right)=-\sum_{i=1}^{N}\alpha_{i}\log\left(1+\gamma_{i}\right) \end{array}$$

where θ denotes the neural network parameters. The loss function is computed by neural network input $\hat{H}$ and output $f\left(\theta ;\hat{H}\right)=W_{nn}$, where f(·) denotes the neural network function. The formulation in (10) applies to the transformer-based NNBF architecture under imperfect CSI, where the SINR terms $\gamma_{i}$ depend on both the true channel H and the estimated channel $\hat{H}$. Consequently, the performance in this setting is contingent upon the proposed network’s ability to effectively handle errors in channel estimation. In contrast, in the case of perfect CSI, as implemented in the simple NNBF architecture, $\gamma_{i}$ depends solely on the true channel H.

To compare the performance of the NNBF output $W_{nn}$, ZFBF $W_{zf}$ and MMSE beamforming $W_{mmse}$ are considered as baseline techniques. These baseline weights are derived from the channel estimate $\hat{H}$ and the noise variance $\sigma^{2}$ as,

(11) $$\begin{array}{cc} W_{zf} & ={\left({\hat{H}}^{H}\hat{H}\right)}^{-1}{\hat{H}}^{H} \end{array}$$

(12) $$\begin{array}{cc} W_{mmse} & ={\left({\hat{H}}^{H}\hat{H}+\sigma^{2}I_{N}\right)}^{-1}{\hat{H}}^{H} \end{array}$$

In scenarios with imperfect CSI, such as those used in transformer-based NNBF, baselines are determined using the estimated channel $\hat{H}$. For the simple NNBF architecture, which operates under perfect CSI, the baseline techniques are evaluated utilizing the actual channel response H to ensure a fair comparison under identical information conditions.

## 4. Complexity Analysis

In this section, we analyze the computational complexities of the proposed neural networks, namely simple NNBF and transformer-based NNBF, in terms of floating-point operations (FLOPs), as a standard metric for benchmarking inference cost in deep learning models [38,43]. FLOPs quantify the number of arithmetic operations (both multiplications and additions) performed during the forward pass of a neural network, providing a hardware-agnostic assessment of computational cost. Consequently, they are directly related to the fundamental operations of neural network layers, encompassing convolutions, activations, and normalizations [44]. We focus specifically on inference-time complexity, as it directly impacts the feasibility of real-time deployment in practical wireless communication systems.

### 4.1. Preliminaries

To analyze the complexity of our network architectures, it is essential to examine the FLOPs of their fundamental building blocks.

#### 4.1.1. Standard Convolution

In a standard convolutional layer, each output element is computed by applying a set of 2D kernels, corresponding to each input channel, over a local segment of the input. Specifically, for each element in the output tensor of shape $\left(C_{\mathrm{out}},H_{\mathrm{out}},W_{\mathrm{out}}\right)$, the kernel performs a weighted sum over an input patch of shape $\left(C_{\mathrm{in}},K_{h},K_{w}\right)$. This involves applying an individual 2D kernel of size $\left(K_{h},K_{w}\right)$ to each input channel followed by summing the results across channels, resulting in a scalar value per output position and channel. Hence, the total FLOPs for the layer scales linearly with the output volume size as expressed in (13). For an input shape of $\left(C_{\mathrm{in}},H_{\mathrm{in}},W_{\mathrm{in}}\right)$ and kernel filter of shape $\left(C_{\mathrm{out}},C_{\mathrm{in}},K_{h},K_{w}\right)$ with an output shape $\left(C_{\mathrm{out}},H_{\mathrm{out}},W_{\mathrm{out}}\right)$, FLOPs for standard convolution are given below

(13) $$\begin{array}{c} {\mathrm{FLOPs}}_{conv_{s}}=2\times \left(C_{\mathrm{out}}\times H_{\mathrm{out}}\times W_{\mathrm{out}}\right)\times \underset{\mathrm{FLOPs}\mathrm{per}\mathrm{output}\mathrm{location}}{\underset{︸}{\left(C_{\mathrm{in}}\times K_{h}\times K_{w}\right)}} \end{array}$$

Although the factor of 2 in multiplication and addition is typically included to reflect both operations, it does not affect the scaling behavior and can be omitted in relative comparisons.

#### 4.1.2. Grouped Convolutions

Grouped convolutions modify the standard convolution operation by partitioning the input and output channels into *g* non-overlapping groups. Each group processes a specific subset of input channels utilizing a corresponding subset of filters, thereby performing smaller *g* independent convolutions in parallel. This reduces computational complexity and facilitates more efficient parameter utilization.

Considering identical input and output tensor dimensions as in the standard convolution case, the total FLOPs formulation in (13) is modified according to this grouping. In each group, the number of input and output channels is reduced by a factor of *g*, and the convolution is performed independently. Consequently, the total FLOPs for grouped convolution is as follows

(14) $$\begin{array}{cc} {\mathrm{FLOPs}}_{conv_{g}} & =g\times 2\times \left(\frac{C_{\mathrm{out}}}{g}\times H_{\mathrm{out}}\times W_{\mathrm{out}}\right)\times \left(\frac{C_{\mathrm{in}}}{g}\times K_{h}\times K_{w}\right)\\ \; & =\frac{2}{g}\times \left(C_{\mathrm{out}}\times H_{\mathrm{out}}\times W_{\mathrm{out}}\right)\times \left(C_{\mathrm{in}}\times K_{h}\times K_{w}\right) \end{array}$$

As shown in (14), grouping reduces the total number of FLOPs by a factor of *g* compared to a standard convolution with the same input/output shapes.

#### 4.1.3. Batch Normalization and GELU Activation

Both batch normalization (BN) and activation functions, such as GELU, operate in an elementwise fashion, applying a consistent mathematical transformation independently to each element of the input tensor. These operations do not involve learnable weights or sliding windows, and their computational cost scales directly with the number of elements in the input.

Let the input tensor have shape $\left(C_{\mathrm{in}},H_{\mathrm{in}},W_{\mathrm{in}}\right)$. The total number of elements is $C_{\mathrm{in}}\times H_{\mathrm{in}}\times W_{\mathrm{in}}$ and each element requires a fixed number of floating-point operations depending on the function applied.

Both batch normalization and activation function performs operations elementwise. Therefore, FLOPs can be written as follows for an input shape of $\left(C_{\mathrm{in}},H_{\mathrm{in}},W_{\mathrm{in}}\right)$

(15) $$\begin{array}{c} {\mathrm{FLOPs}}_{\mathrm{bn},\mathrm{act}}=\alpha \times \left(C_{\mathrm{in}}\times H_{\mathrm{in}}\times W_{\mathrm{in}}\right) \end{array}$$

where α is a constant representing the number of operations per element.

### 4.2. Baseline Techniques

The computational complexities of traditional beamforming schemes, ZFBF and MMSE beamforming, can be derived from their analytical expressions provided in (11), (12). For a channel matrix $H\in C^{M\times N}$, the main operations are as follows:

1. Computation of the matrix $H^{H}H\in C^{N\times N}$, which requires $O(MN^{2})$;
2. Matrix inversion of the resulting N×N matrix, with complexity $O(N^{3})$;
3. Multiplying the result of step 2 by H, again contributing to $O(MN^{2})$.

Hence, the overall computational complexity for each beamforming computation becomes $O(MN^{2}+N^{3})$. This cost happens O(LK) times because the beamforming weights have to be calculated independently for each OFDM symbol and subcarrier pair (L,K). So, the total complexity for the full resource grid in a MIMO system is $O\left(LK(MN^{2}+N^{3})\right)$. This analytical structure emphasizes that the computational burden of traditional beamforming techniques scales cubically with the number of UEs *N*, thereby becoming prohibitive in large-scale MIMO systems.

### 4.3. Simple NNBF

When we investigate the complexity of the simple NNBF architecture given in Figure 4, the output of the stack of basic blocks (BBs), which consist of 1D convolutional layers followed by GELU and batch normalization, is flattened and passed to a series of fully connected (FC) layers, where the input shape is (B,8NMK). The number of neurons in the first and last FC layers scale with *M*,*N*,*K*. As result, the overall computational complexity of simple NNBF is dominated by FC layers, yielding a complexity of O(MNK).

### 4.4. Transformer-Based NNBF

In this section, we examine the computational complexity of transformer-based NNBF by analyzing its building components. We investigate the architecture by breaking it down into functional modules and measuring the complexity of each component using a bottom-up approach, which facilitates a clear comprehension of how each building block impacts the overall computational cost of inference time.

#### 4.4.1. Convolutional Residual Network

To analyze the complexity of the convolutional residual network given in Figure 5, the input tensor’s shape is considered to be (b,c,h,w)=(B,2MN,L,K). The convolutional residual network consists of grouped convolutional layers, where the number of groups *g* is set to be the minimum of the input and output channel dimensions. Each grouped convolutional layer is followed by batch normalization and GELU activation. Furthermore, a pointwise convolutional layer integrates the input into the output through a residual connection.

When we consider the complexity of the first grouped convolutional layer by using (14) and (15), the FLOPS required for the first grouped convolutional layer with an output tensor of shape (B,16MN,L,K) and kernel size $\left(K_{h},K_{w}\right)=\left(3,3\right)$ can be written as

(16) $$\begin{array}{c} O\left(\frac{C_{\mathrm{out}}H_{\mathrm{out}}W_{\mathrm{out}}C_{\mathrm{in}}K_{h}K_{w}}{g}\right)=O\left(MNLK\right) \end{array}$$

where $g=\min(C_{\mathrm{in}},C_{\mathrm{out}})$. This shows that grouped convolution scales linearly with respect to the spatial and channel dimensions.

The subsequent grouped convolutional layers maintain the same asymptotic complexity, since the convolutional residual network consists of a symmetric expansion and compression in channel dimension, with the output channel dimensions for each layer being [2MN,16MN,32MN,32MN,16MN,2MN].

In addition, the pointwise convolutional layer in the residual connection has both input and output shapes of (2MN,L,K) when the kernel size is $\left(K_{h},K_{w}\right)=\left(1,1\right)$. Using (13), its complexity becomes

(17) $$\begin{array}{c} O\left(C_{\mathrm{out}}\times H_{\mathrm{out}}\times W_{\mathrm{out}}\times C_{\mathrm{in}}\times K_{h}\times K_{w}\right)=O\left(M^{2}N^{2}LK\right) \end{array}$$

Then, the overall inference time computational complexity of convolutional residual network can be written as

(18) $$\begin{array}{c} O(MNLK+M^{2}N^{2}LK) \end{array}$$

#### 4.4.2. Stacked Multi-Channel Attention Module

For the complexity analysis of the multi-channel attention module shown in Figure 6, we consider the input tensor’s shape for each attention mechanism to be $\left(b,c,h,w\right)=\left(B,\frac{\dim}{n_{\mathrm{channels}}},L,K\right)$. This module comprises several key components, including positional encoding, embedding layer, self attention transformer, and cross attention transformer. The computational complexity of the stacked multi-channel attention module will be evaluated by examining each of these components.

#### Positional Encoding

The input shape for positional encoding is considered as ${\tilde{X}}_{i}^{0}\in R^{hw\times c}$, where hw corresponds to the sequence length, that is, the flattened dimensions. The positional encoding operation involves a straightforward addition, which is linear in sequence length and feature dimension. The cost of the addition of positional encoding is $O\left(h\times w\times c\right)=O\left(\frac{LK}{n_{\mathrm{channels}}}\right)$.

#### Embedding Layer

The embedding layer consists of 1×1 2D convolutional layer followed by batch normalization. The convolutional layer has a complexity of $O\left(h\times w\times c^{2}\right)=O\left(\frac{LK}{n_{\mathrm{channels}}^{2}}\right)$, when the number of input and output channels is *c* and the spatial dimensions of the output are (h,w). Batch normalization has a complexity of $O\left(h\times w\times c\right)=O\left(\frac{LK}{n_{\mathrm{channels})}}\right)$. Then, the overall embedding layer complexity is

(19) $$\begin{array}{c} O\left(h\times w\times c^{2}+h\times w\times c\right)=O\left(\frac{LK}{n_{\mathrm{channels}}^{2}}+\frac{LK}{n_{\mathrm{channels})}}\right) \end{array}$$

#### Self Attention Transformer

The self attention transformer includes two sublayers. The first sublayer computes attention with the following three steps: projections to query, key, and value tensors (Q,K,V); computation of scaled dot-product attention; and the application of attention scores to values. The second sublayer has two fully connected (FC) layers and GELU activation.

In the first sublayer, considering that the number of heads is indicated by $h_{\mathrm{heads}}$ and the head dimension is $d_{\mathrm{head}}=\frac{c}{h_{\mathrm{heads}}}$, the (query, key, value) projections have three parallel convolutional projections with kernel size 1×1. The convolutional projections are followed by GELU activations. The computational cost of projections are as follows

(20) $$\begin{array}{c} \underset{\mathrm{convolutional}\mathrm{projections}}{\underset{︸}{O(h\times w\times c^{2})}}+\underset{\mathrm{GELU}}{\underset{︸}{O(h\times w\times c)}}=O\left(\frac{LK}{n_{\mathrm{channels}}^{2}}\right)+O\left(\frac{LK}{n_{\mathrm{channels}}}\right) \end{array}$$

When query Q, key K, and value V has the shape of $hw\times d_{\mathrm{head}}$, the scaled dot-product attention complexity is

(21) $$\begin{array}{c} O\left(hw\times d_{\mathrm{head}}\times hw\right)=O\left(\frac{L^{2}K^{2}}{n_{\mathrm{channel}}\times h_{\mathrm{head}}}\right) \end{array}$$

which corresponds to the complexity of the matrix multiplication between query and key. Softmax over dot-product $QK^{T}\in R^{hw\times hw}$ has a complexity of $O\left(h^{2}w^{2}\times h_{\mathrm{head}}\right)=O\left(L^{2}K^{2}\times h_{\mathrm{head}}\right)$. Similarly to dot-product complexity, the application of attention scores to values has a complexity

(22) $$\begin{array}{c} O\left(h^{2}w^{2}\times d_{\mathrm{head}}\right)=O\left(\frac{L^{2}K^{2}}{n_{\mathrm{channel}}\times h_{\mathrm{head}}}\right) \end{array}$$

which corresponds to the matrix multiplication of attention scores with values. The overall complexity of the first sublayer can be written as follows

(23) $$\begin{array}{c} \underset{\mathrm{QKV}\mathrm{projections}}{\underset{︸}{O(hwc^{2})}}+\underset{GELU}{\underset{︸}{O(hwc)}}+\underset{\mathrm{scaled}\mathrm{dot}-\mathrm{product}}{\underset{︸}{O(h^{2}w^{2}\times d_{\mathrm{head}})}}=O\left(\frac{LK}{n_{\mathrm{channels}}^{2}}+\frac{LK}{n_{\mathrm{channels}}}+\frac{L^{2}K^{2}}{n_{\mathrm{channel}}\times h_{\mathrm{head}}}\right) \end{array}$$

In the second sublayer of the self attention transformer, the fully connected layer FC(c,4c) is succeeded by GELU activation and another fully connected layer FC(4c,c). Then, the computational complexity of the second sublayer is

(24) $$\begin{array}{cc} \; & O\left(hw\times c\times 4c\right)=O\left(\frac{LK}{n_{\mathrm{channel}}^{2}}\right),\;\mathrm{FC}(c,4c) \end{array}$$

(25) $$\begin{array}{cc} \; & O\left(hw\times 4c\right)=O\left(\frac{LK}{n_{\mathrm{channel}}}\right),\;\mathrm{GELU} \end{array}$$

(26) $$\begin{array}{cc} \; & O\left(hw\times 4c\times c\right)=O\left(\frac{LK}{n_{\mathrm{channel}}^{2}}\right),\;\mathrm{FC}(4c,c) \end{array}$$

The complexity of the second sublayer can be written as

(27) $$\begin{array}{c} O\left(hwc^{2}+hwc\right)=O\left(\frac{LK}{n_{\mathrm{channel}}^{2}}+\frac{LK}{n_{\mathrm{channel}}}\right) \end{array}$$

The self attention transformer’s overall complexity is

(28) $$\begin{array}{c} O\left(hwc^{2}+hwc+h^{2}w^{2}\times d_{\mathrm{head}}\right)=O\left(\frac{LK}{n_{\mathrm{channels}}^{2}}+\frac{LK}{n_{\mathrm{channels}}}+\frac{L^{2}K^{2}}{n_{\mathrm{channel}}\times h_{\mathrm{head}}}\right) \end{array}$$

#### Cross Attention Transformer

The complexity of the cross attention transformer remains equivalent to that of the self attention transformer, as it merely utilizes distinct inputs.

#### Multi-Channel Attention

The complexity of multi-channel attention is equivalent to the complexities of self attention transformers and cross attention transformers multiplied by $n_{\mathrm{channels}}$, as it consists of self attention and cross attention transformers for $n_{\mathrm{channels}}$ inputs operating in parallel

(29) $$\begin{array}{c} n_{\mathrm{channels}}\times O\left(hwc^{2}+hwc+h^{2}w^{2}\times d_{\mathrm{head}}\right)=O\left(\frac{LK}{n_{\mathrm{channels}}}+LK+\frac{L^{2}K^{2}}{h_{\mathrm{head}}}\right) \end{array}$$

Consequently, the stacked multi-channel attention module is a concatenation of multiple multi-channel attention modules after $n_{\mathrm{channels}}$ positional encodings and $n_{\mathrm{channels}}$ embedding layers. Then, the complexity of stacked multi-channel attention module is

(30) $$\begin{array}{cc} \; & n_{\mathrm{channels}}\times O\left(\frac{LK}{n_{\mathrm{channels}}}\right)+n_{\mathrm{channels}}\times O\left(\frac{LK}{n_{\mathrm{channels}}^{2}}+\frac{LK}{n_{\mathrm{channels})}}\right)+\\ \; & +D\times O\left(\frac{LK}{n_{\mathrm{channels}}}+LK+\frac{L^{2}K^{2}}{h_{\mathrm{head}}}\right)\\ \; & =O\left(\frac{LK}{n_{\mathrm{channels}}}+LK+\frac{L^{2}K^{2}}{h_{\mathrm{head}}}\right) \end{array}$$

where *D* is the number of multi-channel attention modules to be concatenated. Assuming a single head ($h_{\mathrm{head}}=1$), the result is $O\left(\frac{LK}{n_{\mathrm{channels}}}+LK+L^{2}K^{2}\right)$

### 4.5. Summary of Complexity Analysis

A summary of computational complexity analysis is presented in Table 2. The overall complexity of the transformer-based NNBF is mainly driven by two components:

- The pointwise convolution in the convolutional residual network, which incurs a complexity of $O(M^{2}N^{2})$, resulting from quadratic scaling with the number of antennas;
- The scaled dot-product attention and the subsequent multiplication of attention scores with values introduce a complexity of $O(L^{2}K^{2})$, indicating quadratic growth in the OFDM grid size.

These components dominate the inference-time cost and are responsible for the asymptotically quadratic complexity behavior observed in transformer-based NNBF.

**Figure 7:**
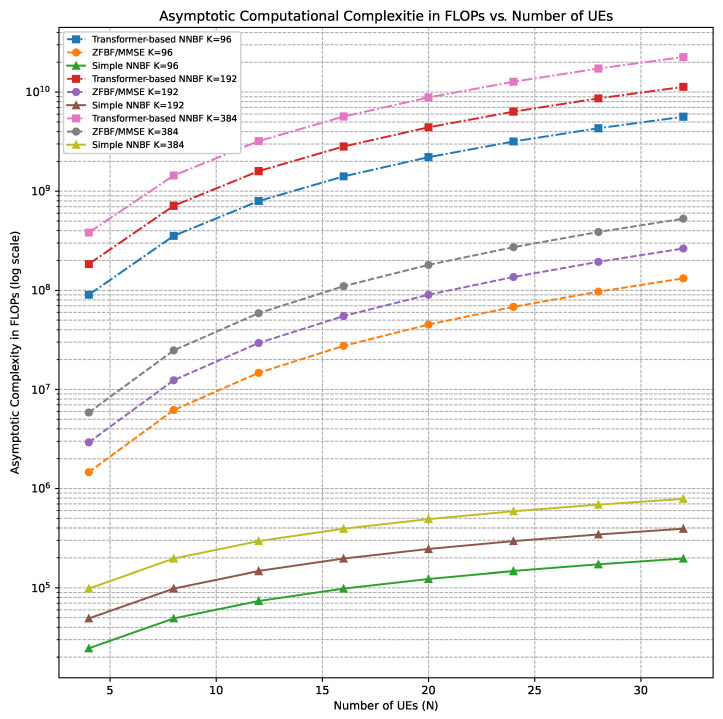
Asymptotic computational complexity (in FLOPs) versus number of UEs *N* for different subcarrier configurations K={96,192,384}. The analysis compares ZFBF/MMSE, simple NNBF, and transformer-based NNBF architectures under fixed receive antenna count M=64 and OFDM symbol length L=14.

In addition, baseline beamforming complexity results exhibit cubic scaling with respect to the number of single-antenna UEs *N*, mainly due to the matrix inversion. In contrast, the simple NNBF maintains linear complexity in *N*, *M*, and *K*, due to its lightweight architecture composed of only convolutional and FC layers. The reduced complexity of the simple NNBF compared to the baseline methods is also demonstrated by Figure 11, which illustrates the GPU runtime in milliseconds as a function of varying *N* for a constant *M* in the next section. While the baseline methods show a noticeable increase in runtime as *N* increases from 4 to 16, the runtime for simple NNBF remains nearly constant. However, it should be noted that the figure displays empirical GPU runtime in milliseconds, which do not directly measure the FLOPs but are affected by hardware-related factors such as GPU parallelism, memory access patterns, and software optimizations. The absence of apparent cubic scaling in ZFBF and MMSE is likely due to the limited scale range of *N* and the highly optimized matrix operations on GPU in that experiment. To further investigate the theoretical scaling trends, asymptotic complexity in terms of FLOPs for a broader range of *N* and *K* values can be seen in Figure 7. While ZFBF and MMSE scale cubically with *N*, the simple NNBF grows linearly. Transformer-based NNBF shows higher complexity dominated by $O\left(M^{2}N^{2}LK\right)$ and exhibits quadratic growth in *K*, reflecting sensitivity to the OFDM grid size.

When comparing transformer-based NNBF with simple NNBF, we observe that a key trade-off emerges between modeling capability and computational efficiency. The transformer-based NNBF benefits from the ability to capture long-range dependencies through attention mechanisms, by making it more expressive for complex spatial and spectral correlations in the MIMO OFDM system. However, it comes with increased computational complexity, particularly due to operations like scaled dot-product attention computation, the application of attention scores on value projections, and pointwise residual convolutions, which incurs quadratic terms in asymptotical complexity. Unlike transformer-based NNBF, simple NNBF achieves significantly lower complexity by relying on lightweight 1D convolutional layers and FC layers. However, the simplifying assumptions, such as stationary of UEs, and availability of perfect CSI, make the simple NNBF useful for only simple scenarios, while it performs poorly in more realistic and dynamical scenarios.

## 5. Experiments

In this section, we evaluate the performance of the proposed NNBF architectures, simple NNBF and transformer-based NNBF, compared to conventional baseline methods, ZFBF and MMSE beamforming. The assessment emphasizes spectral efficiency as a shared throughput-related metric in both models. Additionally, the computational time complexity of the simple NNBF architecture is evaluated to demonstrate its scalability in massive MIMO environments due to its lightweight design, while BLER of the transformed-based NNBF is analyzed to assess its robustness under challenging channel conditions and imperfect CSI.

### 5.1. Experiments with Simple NNBF Architecture

#### 5.1.1. System and Dataset Specifications

Channel responses for the training and evaluation of simple NNBF are generated according to the TDL-A channel model specified by 3GPP TR 38.901 [27]. We utilize four resource blocks, each comprising 12 subcarriers, for system specifications. The maximum Doppler shift for the simple NNBF architecture is established at 10 Hz, assuming slow fading across time slots. Experiments are performed within the SNR range of [−15, 35] dB. The system specifications are presented in Table 3.

#### 5.1.2. Model and Training Details

In our experiments, we select a learning rate of $10^{-4}$. We employ a learning rate scheduler that halves the current learning rate if the validation loss does not improve for three epochs. We employ the AdamW optimizer. The rate weight $\alpha_{i}$ in (10) is defined as $\frac{1}{N}$ for all i=1,…,N. The batch size is set to 8, with the training set comprising 100 batches and the test set consisting of 25 batches per epoch.

#### 5.1.3. Results and Analysis for Simple NNBF

**Figure 8:**
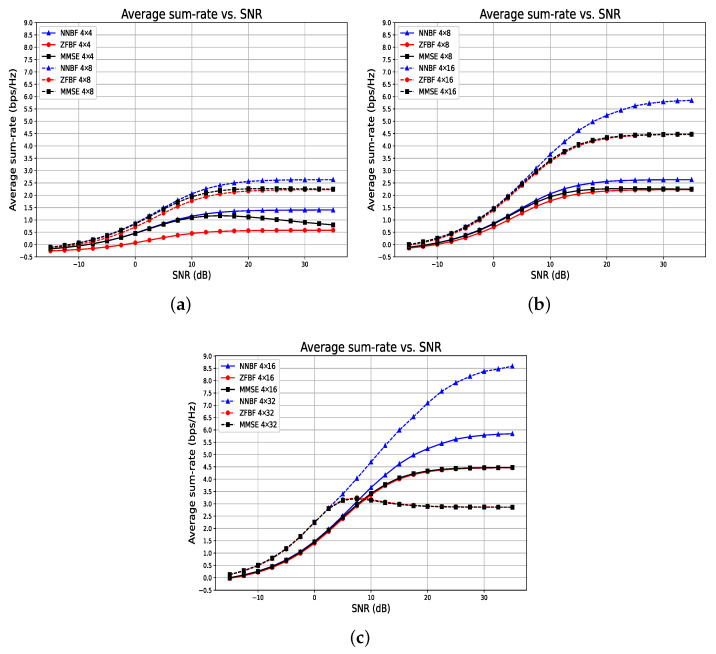
Performance comparison of the simple NNBF model with baseline methods ZFBF and MMSE, with a fixed number of UEs at four and varying numbers of receive antennas across Rx = {4, 8, 16, 32}. Each subplot presents pairwise comparisons as the number of receive antennas increases: (**a**) 4×4 vs. 4×8, (**b**) 4×8 vs. 4×16, (**c**) 4×16 vs. 4×32.

Our experimental analysis utilizing a simple NNBF demonstrates, as shown in Figure 8, the effect of the number of receive antennas on the proposed framework and baseline methods. The results for MMSE, ZFBF, and NNBF are represented by a black square, red circle, and blue triangle, respectively. In all examined scenarios, the quantity of single-antenna UEs is constant at N=4, while the number of receive antennas can be M=4,8,16,32. In the low SNR regime, NNBF exhibits performance comparable to MMSE, whereas ZFBF demonstrates inferior results, as shown by the comparison of the 4×4 and 4×8 scenarios. With an increase in the number of receive antennas, ZFBF can achieve performance levels comparable to those of NNBF and MMSE. In a high SNR regime, MMSE and ZFBF converge to identical outcomes as anticipated, while the proposed framework NNBF significantly outperforms both in all scenarios.

**Figure 9:**
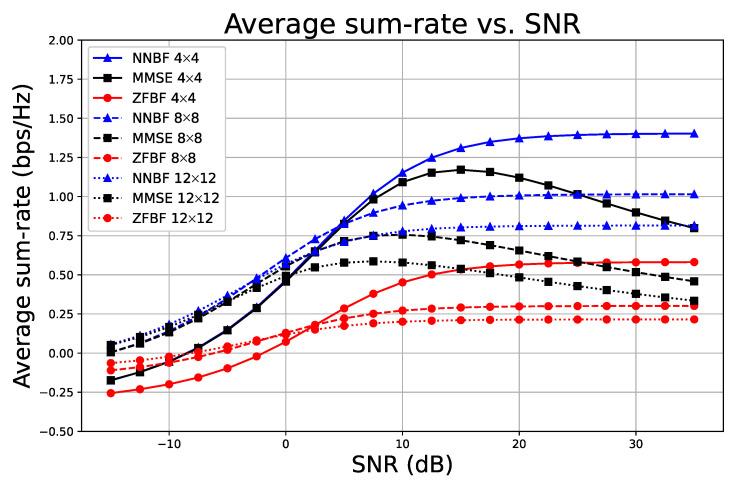
Average sum-rate versus SNR for systems with a fixed 1:1 ratio of receive antennas to single-antenna UEs, where larger systems benefit from spatial diversity at low SNR but suffer from increased interference at high SNR.

Figure 9 presents another experimental configuration in which the ratio of single-antenna user equipment to receive antennas is maintained at a constant 1:1 ratio. Although a 1:1 ratio may lack practical applications and 1:4 ratio scenarios are more prevalent, it is valuable to examine this configuration to facilitate performance comparisons under hardware resource constraints.

In Figure 9, solid lines denote the case for N=4 and M=4, dashed lines indicate the case for N=8 and M=8, and dotted lines represent the case for N=12 and M=12. In the low SNR regime, an increase in the number of antennas enhances spatial diversity, thus improving the mitigation of fading effects. In the high SNR regime, the primary challenge is interference cancelation rather than noise mitigation. While it is anticipated that increasing the number of antennas enhances the spatial degree of freedom, thereby improving spatial multiplexing gain to mitigate interference, an increase in antennas does not inherently guarantee superior performance, particularly when the ratio remains constant. In particular cases involving highly correlated channels, interference cancelation becomes more challenging as the number of UEs grows. Consequently, the 4×4 configuration exhibits the best performance, whereas the 12×12 configuration demonstrates the poorest performance in a high SNR regime, opposing the expected trend. Overall, NNBF outperforms both MMSE and ZFBF across all configurations and SNR ranges, while NNBF also presents reduced performance degradation.

**Figure 10:**
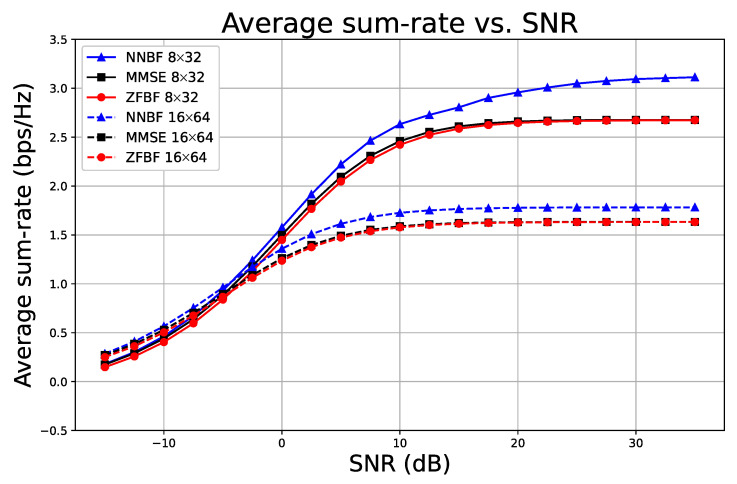
Average sum-rate versus SNR for systems with a fixed 1:4 ratio of receive antennas to single-antenna UEs.

Figure 10 depicts the outcome of a similar experimental configuration, maintaining a constant ratio of single-antenna user equipment to received antennas at 1:4. This is intended to determine if the results of a 1:1 ratio experimental setup are applicable to a 1:4 ratio as well. In Figure 10, solid lines denote the 8×32 configuration, whereas dashed lines indicate the 16×64 configuration. Consistent with the findings in Figure 9, the 16×64 configuration yields superior outcomes in the low SNR regime, whereas the 8×32 configuration demonstrates higher performance in the high SNR domain. NNBF exhibits superior performance compared to the baseline methods across the entire SNR spectrum.

**Figure 11:**
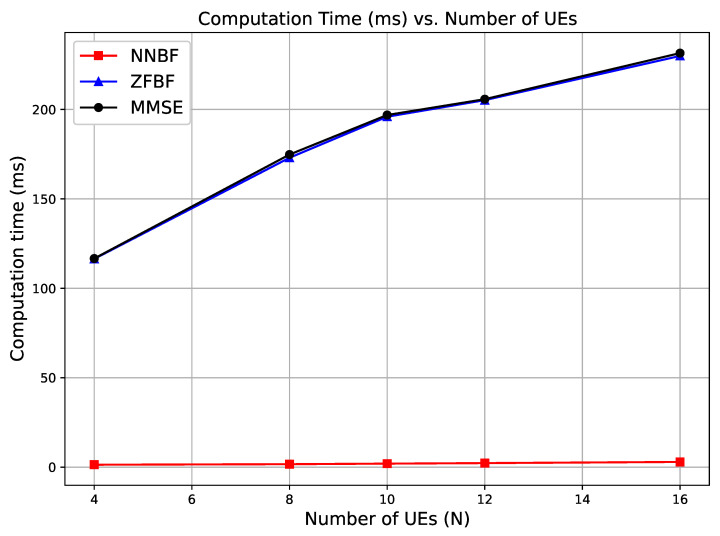
Computation time (ms) versus number of UEs when the number of receive antennas is 64.

Finally, we examine the computational time of the proposed simple NNBF model in comparison to the baseline methods MMSE and ZFBF in this section. Figure 11 illustrates the computation time relative to the number of single-antenna UEs when the number of receive antennas is 64. The computation times for ZFBF and MMSE are comparable, as the complexity of the pseudo-inverse operation predominates the overall computation time. The computation time for NNBF scales with the increasing number of UEs, exhibiting an acceptable growth.

### 5.2. Experiments with Transformer-Based NNBF Architecture

#### 5.2.1. System and Training Specifications

Transformer-based NNBF experiments are performed for antenna configurations of 2×8 and 2×16 as antenna N×M. Model trainings are conducted over a broad SNR range of [−10, 20] dB, encompassing both low and high SNR domains in wireless networks. Channel responses are generated using the UMa channel model with the NVIDIA Sionna library [45], in accordance with 3GPP TR 38.901 specifications [27]. For each batch generation during for both training and evaluation, a new random network topology is generated, with UE positions uniformly distributed within the UMa cell and UE velocities randomly sampled from [0, 30] m/s, ensuring diverse mobility conditions. Hyperparameter optimization for model training is conducted on optimizers {Adam, AdamW, Radam, RMSprop, Adagrad, Adadelta} and learning rate schedulers {ReduceLROnPlateau, CosineAnnealing, CosineAnnealingWarmRestarts, ExponentialLR, CyclicLR} in Optuna [46]. The Lookahead optimizer is employed with a base optimizer to enhance convergence speed and stability, using Lookahead steps of k=13 and an update coefficient of $\alpha_{\mathrm{la}}=0.5$. The base optimizer updates model parameters for *k* iterations to derive fast parameters $\theta_{t}^{\mathrm{fast}}$, subsequently updating slow parameters as

(31) $$\begin{array}{c} \theta_{t}^{\mathrm{slow}}=\theta_{t-k}^{\mathrm{slow}}+\alpha_{\mathrm{la}}\left(\theta_{t}^{\mathrm{fast}}-\theta_{t-k}^{\mathrm{slow}}\right) \end{array}$$

A curriculum learning strategy is employed, wherein training advances from simpler to more intricate tasks by modifying the signal-to-noise ratio at each phase. The maximum SNR is set at 20 dB, whereas the minimum SNR for each stage is refined via hyperparameter optimization. System and training parameters are summarized in Table 4.

#### 5.2.2. Results and Analysis for Transformer-Based NNBF

**Figure 12:**
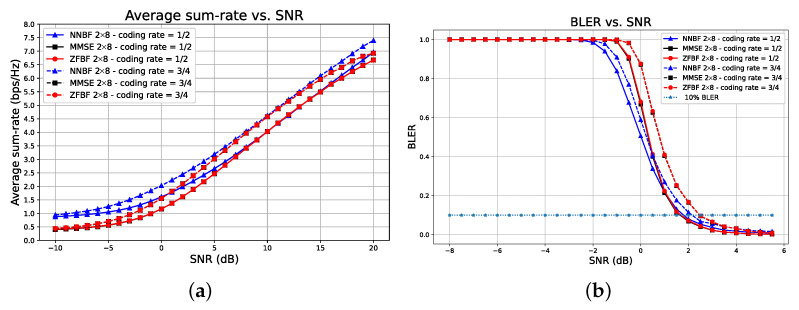
Performance comparison of transformer-based NNBF with baseline methods ZFBF and MMSE for a 2×8 system using 4QAM and stationary UEs, with coding rates of $\frac{1}{2}$ and $\frac{3}{4}$. Transformer-based NNBF achieves higher spectral efficiency across a broad SNR range, even surpassing higher coding rate baselines at low SNR (**a**), while preserving communication reliability with comparable BLER performance (**b**).

Figure 12 depicts the impact of coding rate, that is, the ratio of useful bits to the total bits transmitted, including redundancy. The system configuration is demonstrated for a 2×8 scenario, with the UEs remaining stationary and employing 4QAM modulation. The coding rate may be $\frac{1}{2}$ or $\frac{3}{4}$. In Figure 12a, the average sum-rate metric indicates that transformer-based framework NNBF outperforms baseline techniques within the SNR range of [−10,5] dB, spanning both low and high SNR regimes pertinent to practical wireless network scenarios. Furthermore, transformer-based NNBF with a coding rate of $\frac{1}{2}$ (solid blue triangle line) surpasses the baseline techniques with a coding rate of $\frac{3}{4}$ (dashed black square and dashed circle lines) in the low SNR regime of [−10,−3] dB, despite the expectation that a higher coding rate would yield higher throughput. This result indicates that transformer-based NNBF can achieve greater throughput despite a lower coding rate. Besides spectral efficiency, we can evaluate the transformer-based NNBF framework against baseline techniques regarding communication reliability. Figure 12b displays the BLER performance. Transformer-based NNBF exhibits a lower BLER than MMSE and ZFBF, achieving 10% BLER at similar channel SNR values of [1.5,2.5] dB. Consequently, it demonstrates that the transformer-based framework offers enhanced spectral efficiency while maintaining communication reliability.

**Figure 13:**
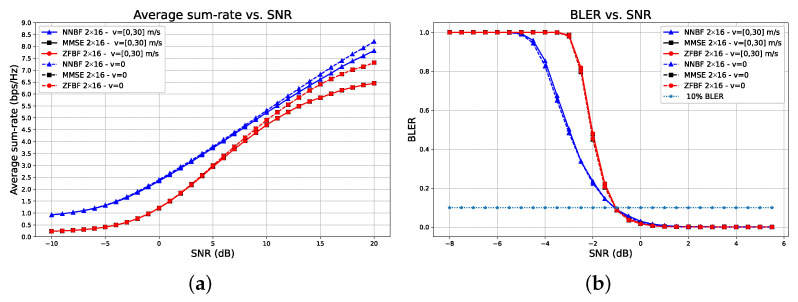
Performance comparison of transformer-based NNBF with ZFBF and MMSE for stationary and mobile UEs (up to 30 m/s) using a 2 × 16 system with 4QAM and coding rate $\frac{1}{2}$. Subfigures show different performance metrics: (**a**) SINR performance and (**b**) BLER performance. Transformer-based NNBF demonstrates robust performance under mobility, significantly outperforming the baselines in mitigating mobility-induced degradation.

Figure 13 presents a performance comparison of transformer-based NNBF against ZFBF and MMSE beamforming for both stationary and mobile UEs. Mobile UEs can reach velocities of up to 30 m/s. The system configuration is provided for a 2×16 setup utilizing 4QAM modulation, with a coding rate established at $\frac{1}{2}$. In mobile communications, the Doppler shift frequency $f_{d}=\frac{f_{c}v\cos\phi }{c}$ quantifies the frequency shift of the signal resulting from the motion of the mobile UE, where *c* represents the speed of light, *v* is the velocity of the UE, and ϕ indicates the angle between the BS and the UE. The carrier frequency, denoted by $f_{c}$, is established at 2.6 GHz in our experiments. The maximum Doppler shift for the experiments depicted in Figure 13 is 260 Hz, given that the maximum velocity is 30 m/s. Figure 13 illustrates that the proposed framework, transformer-based NNBF, is capable of handling the impact of user equipment mobility, which is inevitable in urban network environments. In terms of spectral efficiency, we note a significant decrease in average sum-rate after 10 dB for baseline techniques, whereas transformer-based NNBF exhibits only a negligible drop.

**Figure 14:**
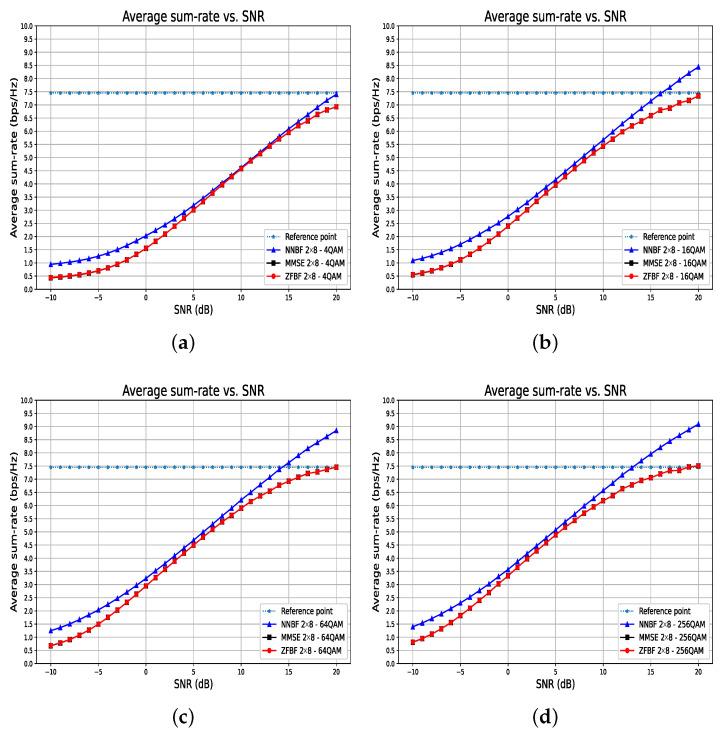
Average sum-rate performance of transformer-based NNBF versus ZFBF and MMSE for increasing modulation orders ((**a**) 4QAM, (**b**) 16QAM, (**c**) 64QAM, (**d**) 256QAM) in a stationary 2 × 8 system with coding rate $\frac{3}{4}$. Transformer-based NNBF benefits more from higher-order modulations, leading to greater spectral efficiency compared to baseline techniques.

**Figure 15:**
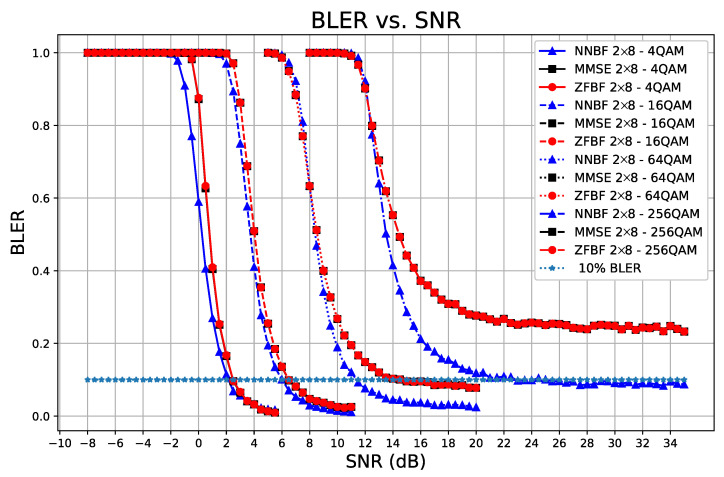
BLER performance of transformer-based NNBF compared to ZFBF and MMSE for increasing modulation orders (4QAM to 256QAM) in a stationary 2 × 8 system with coding rate $\frac{3}{4}$. Transformer-based NNBF shows greater robustness, consistently achieving lower error rates than baseline techniques as modulation complexity increases.

Figure 14 and Figure 15 illustrate the average sum-rate and BLER performance of the transformer-based NNBF compared to baseline techniques for increasing orders of modulation, respectively. Figure 14 illustrates that the average sum-rate of baseline techniques converges to the reference point as the modulation order increases. The average sum-rate for transformer-based NNBF continues to improve with the increase in modulation order, indicating that it may ultimately converge to a greater value. The transformer-based NNBF model effectively utilizes the enhanced spectral efficiency offered by higher order modulation schemes, whereas the baseline techniques are constrained by their suboptimal beamforming strategies. Moreover, in all previous experiment results, comparable BLER performances are observed for transformer-based NNBF and baseline techniques when the modulation type is specified as 4QAM. As the modulation order increases, the superiority of NNBF in terms of BLER performance becomes apparent compared to MMSE and ZFBF, as illustrated in Figure 15. For 64QAM, transformer-based NNBF could achieve 10% BLER at 11.5 dB, whereas MMSE and ZFBF can achieve 10% BLER at 13.5 dB. For 256QAM, MMSE and ZFBF converge to 20% BLER, while transformer-based NNBF can still achieve a BLER below 10%.

## 6. Conclusions

In this work, we presented a unified and systematic comparison of two models of unsupervised deep learning for uplink receive beamforming, namely a simple NNBF model and a transformer-based NNBF model. In addition, we provided a FLOPs-based asymptotic computational complexity analysis to characterize the inference-time scaling behavior of the considered architectures alongside conventional beamforming baseline techniques, ZFBF and MMSE beamforming. The primary objective was the sum-rate maximization of MU-SIMO system with 3GPP-compliant channel models. The simple NNBF, developed under the assumption of perfect CSI and stationary UEs in the TDL-A channel model, demonstrated competitive or superior performance compared to MMSE beamforming, while consistently outperforming the ZFBF performance across all SNR regimes for various antenna configurations. Moreover, both theoretical analysis and empirical results confirmed that simple NNBF offers significantly lower computational complexity compared to the baseline methods. To address more realistic scenarios, the transformer-based NNBF combined grouped convolutions and transformers, enabling robust performance under UMa channels with imperfect CSI and high UE mobility. Extensive simulations across various mobility, coding rate, and modulation configurations confirmed its superiority in terms of spectral efficiency and BLER compared to the baseline methods ZFBF and MMSE beamforming. We specifically noted that the transformer-based NNBF exhibits robust performance under mobility by alleviating mobility-induced degradation. Furthermore, it is demonstrated that transformer-based NNBF exhibits higher throughput from higher-order modulations, resulting in improved spectral efficiency, while consistently achieving lower error rates than the baseline techniques as modulation complexity increases. Furthermore, our theoretical analysis revealed a trade-off between the increased modeling capacity provided by attention mechanisms and the resulting computational cost, which scales quadratically with the OFDM grid size in terms of asymptotic complexity. In contrast to the simplified assumptions of simple NNBF, this additional cost is needed to manage realistic and dynamic channel conditions. The results encourage future research into more efficient neural architectures that are specifically designed for such environments.

## Figures and Tables

**Table 1:** Uplink functional distribution across 7.2x categories and ULPI variants.

Function	Cat-A	Cat-B	ULPI-A	ULPI-B
Cyclic Prefix Removal	O-RU	O-RU	O-RU	O-RU
FFT	O-RU	O-RU	O-RU	O-RU
Channel Estimation	O-DU	O-RU	O-DU	O-RU
Uplink Beamforming	O-DU	O-RU	O-DU	O-RU
Equalization	O-DU	O-DU (optional)	O-DU	Optional (O-RU or O-DU)
Compression	Optional	Optional	Enhanced	Enhanced
Output to O-DU	Antenna-domain IQ	Beamformed UE streams	Compressed IQ	Beamformed or demodulated UE streams
RU Complexity	Low	High	Medium	Very High
Fronthaul Bandwidth	High	Reduced	Lower	Minimal
Latency Requirement	Strict	Relaxed	Relaxed	Most Relaxed

**Table 2:** Complexity analysis.

Technique	Complexity
ZFBF	$O(MN^{2}LK+N^{3}LK)$
MMSE	$O(MN^{2}LK+N^{3}LK)$
Transformer-based NNBF	$O\left(\frac{LK}{n_{\mathrm{channels}}}+LK+L^{2}K^{2}+MNLK+M^{2}N^{2}LK\right)$
Simple NNBF	O(MNK)

**Table 3:** System parameters for simple NNBF architecture experiments.

Parameter	Value
Channel delay profile	TDL-A
Number of resource blocks (RBs)	4 (48 subcarriers)
Delay spread	30 ns
Maximum Doppler shift	10 Hz
Subcarrier spacing	30 kHz
Transmission time interval (TTI)	500 μs
SNR	[−15, 35] dB
Modulation scheme	QPSK

**Table 4:** System and training parameters for transformer-based NNBF experiments.

Parameter	Value
Number of resource blocks (RBs)	4 (48 subcarriers)
Maximum Doppler shift $f_{d}$	260 Hz
Maximum UE velocity *v*	30 m/s
Carrier frequency $f_{c}$	2.6 GHz
Subcarrier spacing	30 kHz
Transmission time interval (TTI)	500 μs
Coding rate	$\frac{1}{2}$, $\frac{3}{4}$
Modulation scheme	4QAM, 16QAM, 64QAM, 256QAM
Training SNR	[−10, 20] dB
Learning rate	$\left[10^{-5},10^{-2}\right]$
$\alpha_{\mathrm{la}}$	0.5
k	13
Minimum training SNR ranges	[15, 20], [10, 15], [5, 10], [0, 5], [−10, 0]

## Data Availability

The data supporting the findings of this study were generated using the Sionna simulation library and internal proprietary code developed at DeepSig Inc. Due to confidentiality restrictions, the simulation code and full dataset cannot be publicly released.
